# Improving Fucoxanthin Production in Mixotrophic Culture of Marine Diatom *Phaeodactylum tricornutum* by LED Light Shift and Nitrogen Supplementation

**DOI:** 10.3389/fbioe.2020.00820

**Published:** 2020-07-15

**Authors:** Runqing Yang, Dong Wei

**Affiliations:** School of Food Science and Engineering, South China University of Technology, Guangzhou, China

**Keywords:** fucoxanthin, *Phaeodactylum tricornutum*, light regime, two-phase culture, qRT-PCR

## Abstract

Fucoxanthin (Fx), a kind of primary carotenoids in brown seaweeds and diatoms, has attractive efficacy in human’s healthcare including loss weight, the prevention of diabetes and Alzheimer’s disease. Marine diatom *Phaeodactylum tricornutum* is now realized as a promising producer for commercial Fx production due to its higher content of Fx than brown seaweeds with easily artificial cultivation and Fx extraction. In the present study, to improve Fx production in *P. tricornutum*, the mixotrophic cultures were applied to optimize initial cell density, light intensity, light regime and nitrogen supplementation. The results showed that the higher initial cell density (1 × 10^7^ cells mL^–1^) and lower light intensity (20 μmol m^–2^ s^–1^) were favorable for biomass production and Fx accumulation. The maximal Fx content [16.28 mg g^–1^ dry weight (DW)] could be achieved under blue light (BL), but the highest biomass concentration (5.53 g L^–1^) could be attained under red: blue light (R: B, 6:1) in the batch culture. A novel two-phase culture approach was developed to increase the biomass concentration to the highest value (6.52 g L^–1^) with the maximal productivity of Fx (8.22 mg L^–1^ d^–1^) through light shift from R:B ratio (6:1) in phase 1 to R:B ratio (5:1) by enhancing BL and tryptone addition in phase 2. The content and intracellular amount of Fx were also increased 8% and 12% in phase 2 compared to phase 1. The expression levels analysis revealed that genes encoding phytoene synthase (PSY), zeaxanthin epoxidase (ZEP), and fucoxanthin-chlorophyll-protein b (FCPb) were upregulated significantly, with downregulation of the gene encoding violaxanthin de-epoxidase (VDE), leading to the improvement of Fx in phase 2. The present study demonstrated the two-phase culture strategy could promote Fx productivity through enhancing biomass production and increasing Fx content, indicating that strengthening BL coupled with adding tryptone were effective to facilitate Fx production by mixotrophic cultivation of marine diatom *P. tricornutum*.

## Introduction

Fucoxanthin is a kind of primary carotenoids and draws increasing attention because of its functions of anti-oxidant, anti-obesity, and anti-cancer as well as effects against Alzheimer’s disease (Vilchez et al., 2011; Fu et al., 2015; Xiang et al., 2017). The commercial source of Fx is mainly from brown seaweeds, which are difficult to meet the market demands due to the low productivity, low quality and high cost. Currently, marine diatom *Phaeodactylum tricornutum* is realized as a promising producer for commercial production of Fx since it grows fast and contains higher amount of Fx [1% to 6% of dry weight (DW)], which is over one hundred-fold of brown seaweeds (Rajauria et al., 2017; McClure et al., 2018). *P. tricornutum* is usually cultivated in deep tanks and open ponds for aquaculture, as well as in artificial photobioreactors recently for industrial purpose under autotrophic mode (Gao et al., 2017; Delbrut et al., 2018). Importantly, most strains of *P. tricornutum* could use glycerol and urea as organic carbon and nitrogen source, and biomass could reach to 3∼15 g L^–1^ under mixotrophic mode (Garcia et al., 2005; Huang et al., 2015; Nur et al., 2019). However, the mixotrophic *P. tricornutum* has not been applied in large-scale cultivation system because of the technological barriers mainly on the big risk of contamination when use organic nutrients in open system (Matsumoto et al., 2017). Compared to the autotrophic culture, the mixotrophic culture enhanced the cell growth rate and biomass production, but reduced the photosynthesis activity, leading to the decreased content of photosynthetic pigments (Liu et al., 2009). The content of carotenoids in *P. tricornutum* under mixotrophic conditions was usually 0.5∼0.7% of DW, in which Fx portion was even lower (Ceron-Garcia et al., 2013; Patel et al., 2019). It seems difficult to achieve high biomass and high Fx content simultaneously, resulting in the low productivity of Fx by mixotrophic *P. tricornutum*. Therefore, developing an applicable approach of mixotrophic *P. tricornutum* is vital to commercial production of Fx.

Light regime, including light intensity, light quality and light/dark cycle, is indispensable in the mixotrophic cultivation. In *P. tricornutum*, Fx binds with Chl *a* + *c* and proteins to form FCP complex (Durnford et al., 1999), playing an important role in light harvesting and non-photochemical quenching (NPQ) (Havurinne and Tyystjarvi, 2017; Wang et al., 2019). Different from other microalgae, the specific structure of FCP in *P. tricornutum* allows it to capture blue-green light and supports its application in artificial cultivation system (Wang et al., 2019). At present, the impacts of light quality (presented as light spectrum) on *P. tricornutum* were usually investigated under autotrophic mode. For example, red light could promote cell growth and blue light (BL) could enhance Fx accumulation (Sirisuk et al., 2018; Wang S. et al., 2018). Also, blue LED light could save 50% and 75% energy input compared to red-blue LED light and white fluorescent light, respectively, which is beneficial for industrial use (Wang S. et al., 2018). Under green light, the intracellular amount of Fx was similar with white light (WL), while the ratio of FCP in thylakoid membrane proteins was significantly increased (Zhao, 2015). Accordingly, a two-phase culture with different light regimes has been used for promoting biomass and Fx production. For example, a hetero-photoautotrophic two-phase cultivation with white: blue light (1:1) induction was used in marine diatom *Nitzschia laevis* for Fx accumulation (Lu et al., 2018). Two-phase culture using different light regimes has also applied in inducing lipid accumulation in *P. tricornutum* (Sirisuk et al., 2018; Jung et al., 2019). It is noteworthy that light is not only energy source in the mixotrophic culture of microalgae, but also a role of inducing factor in accumulation of biomass and bioactive compounds in diatom. So far, it is still lack of reports for improving Fx production by mixotrophic *P. tricornutum* under different light regimes.

The whole genome sequencing of *P. tricornutum* was completed in 2008, which provides the biological basis for transcriptome analysis of gene expression and regulation (Bowler et al., 2008). Transcriptome and metabolome analysis indicated that the central-carbon metabolism, especially glycolysis, was enhanced by glycerol, the organic carbon source used in the mixotrophic mode, leading to the increase of cell growth rate and the final cell density (Villanova et al., 2017). The expression levels of genes involving Fx biosynthesis were investigated in recent years, but the reports are rare. For instance, the genetic engineering study demonstrated that the overexpression of *DXS* and *PSY* genes could result in the significantly increase of Fx content (Kadono et al., 2015; Eilers et al., 2016). Under autotrophic condition, the most of genes involving Fx biosynthesis, including *PSY*, *PDS*, *ZDS*, *LCYB*, and *ZEP* were upregulated by blue or green light (Coesel et al., 2008; Valle et al., 2014). It is still very limited to know the regulation of genes expression in Fx biosynthesis pathway under different light regimes and two-phase culture mode under mixotrophic conditions.

In the present work, the mixotrophic *P. tricornutum* was cultivated in shake flasks to optimize the growth conditions for improving Fx production. Initial cell density, LED light intensity and light quality were firstly investigated to obtain optimal parameters, subsequently a two-phase culture approach was developed for promoting Fx productivity by LED light shift and nitrogen supplementation. The expression levels of several key genes in Fx biosynthesis pathway were analyzed by quantitative real time polymerase chain reaction (qRT-PCR), aiming to reveal the metabolic regulation in two-phase culture process.

## Materials and Methods

### Microalgal Strain and Seed Culture

Marine diatom *P. tricornutum* CCMP 1327 was kindly provided by Dr. Hanhua Hu in Institute of Hydrobiology, Chinese Academy of Sciences, Wuhan, China. The seed culture was applied in 250-mL Erlenmeyer flasks containing 100-mL modified f/2 medium (Guillard, 1975) under mixotrophic condition. Temperature at 20°C was setup with continuous illumination of 10 μmol m^–2^ s^–1^ under white LED light and rotating speed of 150 r/min in shaking incubator. The modified f/2 medium contained (per liter): 20 g sea salt, 9.20 g glycerol (0.10 mol L^–1^), 10 mg NaH_2_PO_4_.H_2_O, 30 mg Na_2_SiO_3_.9H_2_O, 3.15 mg FeCl_3_.6H_2_O, 4.36 mg Na_2_EDTA.2H_2_O, 9.80 μg CuSO_4_.5H_2_O, 6.30 μg Na_2_MoO_4_.2H_2_O, 22 μg ZnSO_4_.7H_2_O, 10 μg CoCl_2_.6H_2_O, 180 μg MnCl_2_.4H_2_O. In addition, 1.17 g L^–1^ of tryptone and 0.30 g L^–1^ of urea (1:1, N mol/N mol) were added into the medium to final concentration of total nitrogen (TN) at 0.02 mol L^–1^. The medium was autoclaved at 121°C for 20 min followed by urea addition using stock solution filtered through 0.45 μm membrane. The seed culture in late logarithmic phase was harvested by centrifugation and re-suspended in the medium above as the inoculum for subsequent experiments.

### Optimization of Initial Cell Density and Light Intensity

A series of initial cell densities (1 × 10^6^, 4 × 10^6^, 7 × 10^6^, 1 × 10^7^ cells mL^–1^) were investigated by adding the inoculum and culturing for 14 days. Then, various light intensities (10, 20, 30, 40, 50, 100, 150, 200 μmol m^–2^ s^–1^) were evaluated at initial cell density of 1 × 10^7^ cells mL^–1^ by cultivation for 12 days. The other culture conditions were same as the seed culture. Cell density, glycerol and nitrogen concentration were measured every 2 days during the cultivation. Biomass, content and volumetric concentration of Fx were measured at the end of cultivation.

### Optimization of Light Regimes and Nitrogen Supplementation

#### White Light and Red: Blue Light

To explore the effects of different LED light qualities on biomass production and Fx accumulation, the full-spectrum WL and different red: blue lights (R:B, 0:1, 6:1, 1:1, 1:2, 1:0) were evaluated at initial cell density of 1 × 10^7^ cells mL^–1^ under 20 μmol m^–2^ s^–1^ of light intensity. Cell density, biomass, glycerol and nitrogen concentration were measured every 2 days during the cultivation. The content and volumetric concentration of Fx were analyzed at the end of cultivation.

#### Light Shift and Nitrogen Supplementation in Two-Phase Culture

Two-phase culture approach was investigated to promote biomass and Fx production by light shift and nitrogen supplementation through two batch cultures.

In batch 1, R: B light (6:1) at 20 μmol m^–2^ s^–1^ was used in the mixotrophic cultivation for 6 days in phase 1, and then shifted to BL at 20 μmol m^–2^ s^–1^ with tryptone (T), urea (U) or the mixture (T:U = 1:1, N mol) addition in the medium to final concentration of TN (0.02 mol L^–1^) in phase 2. The culture without nitrogen addition in phase 2 was set as the control.

In batch 2, the two-phase culture with light shift to BL and tryptone addition (BL + T) was set as the control. In the experimental groups, R: B light (6:1) at 20 μmol m^–2^ s^–1^ was used to culture for 6 days in phase 1, then BL was strengthened alone to form various R: B lights (5:1, 3:1, 1:1) at 25 μmol m^–2^ s^–1^, or shifted to pure green light (GL) at 20 μmol m^–2^ s^–1^ with tryptone addition in phase 2. The cell growth and nutrient consumption were monitored every 2 days during the cultivation. Fx production were analyzed at the end of culture. The cells in the culture of R: B light (5:1) at 25 μmol m^–2^ s^–1^ on the 6th, 8th, 10th, 12th day were collected for Fx detection and total RNA isolation.

### Analytical Methods

#### Cell Growth and Biomass Concentration

Cell density was determined by CytoFLEX flow cytometry (Beckman-Coulter, United States). Specific growth rate (μ, d^–1^) of cells was calculated by the following formula:

(1) $$\mu \,\left(d^{-1}\right)=\left({\text{lnN}}_{\text{t}}-{\text{lnN}}_{0}\right)/\left(\text{t}-{\text{t}}_{0}\right)$$

where N_t_ and N_0_ are the cell density (cells mL^–1^) at time t(d) and time t_0_ (d) (Chen et al., 2017).

2-mL cell suspension was collected in a pre-weighed tube and centrifuged at 3300 × g for 3 min. The pellet was washed twice and dried in a 60°C oven to a constant weight for biomass measurement. The biomass productivity (P_Biomass_, mg L^–1^ d^–1^) was calculated by following formula:

(2) $${\text{P}}_{\text{Biomass}}\,\left(\mathrm{mg}\,L^{-1}\,{\text{d}}^{-1}\right)=\left({\text{DW}}_{\text{t}}-{\text{DW}}_{0}\right)/\left(\text{t}-{\text{t}}_{0}\right)\times 1000$$

where DW_0_ and DW_t_ are the biomass concentration (g L^–1^) at time t_0_(d) and time t(d) (Chen et al., 2017).

#### Glycerol and Nitrogen Concentrations

Glycerol concentration (g L^–1^) was determined by M-100 Biosensors (SIEMAN, China). The TN concentration (TN, mg L^–1^) was determined by DR2700 spectrophotometer (HACH, United States) with reagent No. 2714100 (Qin et al., 2018). The nutrient consumption rate (CR, mg L^−1^ d^−1^) and biomass yield per TN consumed (Y_X/TN_, mg mg^–1^) were calculated by following formula:

(3) $$\mathrm{Glycerol}\,\mathrm{CR}\,\left(\mathrm{mg}\,L^{-1}\,{\text{d}}^{-1}\right)=\left({\text{GC}}_{0}-{\text{GC}}_{\text{t}}\right)/\left(\text{t}-{\text{t}}_{0}\right)\times 1000$$

where GC_t_ and GC_0_ are the glycerol concentration (g L^–1^) at time t (d) and time t_0_ (d).

(4) $$\mathrm{TN}\,\mathrm{CR}\,\left(\mathrm{mg}\,L^{-1}\,{\text{d}}^{-1}\right)=\left({\text{NC}}_{0}-{\text{NC}}_{1}+{\text{NC}}_{2}-{\text{NC}}_{\text{t}}\right)/\left(\text{t}-{\text{t}}_{0}\right)$$

where NC_t_ and NC_0_ are the TN concentration (mg L^–1^) at time t(d) and time t_0_(d), NC_1_ and NC_2_ are the TN concentration (mg L^–1^) before and after the nitrogen addition on the 6th day in two-phase cultivation.

$${\text{Y}}_{\text{X}/\text{TN}}\,\left(\text{mg}\,{\text{mg}}^{-1}\right)=\left({\text{DW}}_{\text{t}}-{\text{DW}}_{0}\right)/\left({\text{NC}}_{0}-{\text{NC}}_{1}+{\text{NC}}_{2}-{\text{NC}}_{\text{t}}\right)$$

(5) ×1000

where DW_0_ and DW_t_ are the biomass concentration (g L^–1^) at time t_0_ (d) and time t (d), NC_t_ and NC_0_ are the TN concentration (mg L^–1^) at time t (d) and time t_0_ (d), NC_1_ and NC_2_ are the TN concentration (mg L^–1^) before and after the nitrogen addition on the 6th day in two-phase cultivation.

#### Pigments

Natural pigments were extracted by organic solvents, and the qualitative and quantitative analysis were carried out by high performance liquid chromatography (HPLC) method modified from references (Zang et al., 2015; Chen et al., 2017). Briefly, 10-mg freeze-dried algal powder was mixed with ceramic bead and the mixture of acetone: methanol (1:1, v/v) precooled at 0–10°C in 15-mL tube, and then disrupted using the grinder (Tissuelyser-24, JINGXIN, China) at 70 Hz for 30 s. The supernatant was collected by centrifugation at 5900 × g for 3 min after freezing in the liquid nitrogen for 30 s. The disruption and centrifugation process were repeated until the pellet was colorless, and then dried all supernatants by nitrogen flow gas. 1-mL methanol: methyl tert-butyl ether solution (1:1, v/v) was added to dissolve the residue and filtered through a 0.22 μm nylon membrane for further pigments detection.

High performance liquid chromatography system (DIONEX P680, Thermo Scientific, Waltham, MA, United States) equipped with PDA detector and YMC^TM^ Carotenoids column (150 mm × 4.6 mm, 3 μm) was employed for pigments analysis. The column temperature was maintained at 30°C, the flow rate was 0.8 mL min^–1^, and detection wavelength was 440 nm. Methanol and methyl tert-butyl ether were employed as mobile phases A and B, respectively. The gradient program was as followed: 0∼6 min, A: 95%→80%, B: 5%→20%; 6∼12 min, A: 80%→60%, B: 20%→40%; 12∼19 min, A: 60%→55%, B: 40%→45%; 19∼20 min, A: 55%→95%, B: 45%→5%; 20∼23 min, A: 95%, B: 5%. The peaks of pigments were characterized according to the retention time of Fx and Chl **a** standards (Sigma-Aldrich Chemical Co., St. Louis, MO, United States), and external standard curve was used for quantification. The volumetric concentration of Fx (VC_Fx_, mg L^–1^), intracellular amount of Fx (CC_Fx_, pg cell^–1^) and Fx productivity (P_Fx_, mg L^–1^ d^–1^) were calculated by the following formula:

(6) $${\text{VC}}_{\text{Fx}}\,\left(\mathrm{mg}\,L^{-1}\right)=\text{Biomass}\,\left(g\,L^{-1}\right)\times \mathrm{Fx}\,\mathrm{content}\,\left(\mathrm{mg}\,g^{-1},\text{DW}\right)$$

(7) $${\text{CC}}_{\text{Fx}}\,\left(\mathrm{pg}\,{\mathrm{cell}}^{-1}\right)={\text{VC}}_{\text{Fx}}\,\left(\mathrm{mg}\,L^{-1}\right)/\mathrm{Cell}\,\mathrm{density}\,\left(\mathrm{cells}\,{\mathrm{mL}}^{-1}\right)$$

(8) $${\text{P}}_{\text{Fx}}\,\left(\mathrm{mg}\,L^{-1}\,{\text{d}}^{-1}\right)=\left({\text{VC}}_{\text{Fxt}}-{\text{VC}}_{\text{Fx}\,0}\right)/\left(\text{t}-{\text{t}}_{0}\right)$$

where VC_Fx__0_ and VC_Fxt_ are the volumetric concentration of Fx (mg L^–1^) at time t_0_(d) and time t(d).

#### Quantitative Real Time PCR

To evaluate the expression levels of key genes in Fx biosynthesis pathway during two-phase culture, total RNA isolation from cells under R:B light (5:1) group on the 6th, 8th, 10th, 12th day was carried out using Plant RNA Kit (Omega, America) for qRT-PCR analysis. Evo M-MLV RT Kit for gDNA clean and SYBR Green Premix *Pro Taq* HS qPCR Kit (Accurate biotechnology, China) were employed. qRT-PCR analysis was performed on CFX96 Touch^TM^ Deep Well Real-Time PCR Detection System (Bio-rad, America). The gene coding β-actin was selected as an internal control (Xie et al., 2014). The sequences of target genes were obtained from KEGG database^1^ and primers shown in Table 1 were designed by NCBI.^2^ Total RNA samples were performed in triplicates. The relative expression levels of target gene transcripts were normalized using β-*actin* as reference gene by the 2^−ΔΔCt^ method (Livak and Schmittgen, 2001).

**TABLE 1:** Primer sequence of key genes in fucoxanthin biosynthesis pathway.

**Gene**	**Primer (5′-3′)**	**Gene**	**Primer (5′-3′)**
β*-actin*	GACTCCACCTTCCAGACCATTA	*LCYB*	GCATTGCGACGTACATGGTC
	GACCCTCCAATCCAAACAGAG		TCGTCGAGCTTCACTCTTGG
*DXS*	AGCCAATTCTGGACTCGGTG	*ZEP1*	GGCACTCGAACGCATCAATC
	GCAAGGCAACAGTGAGTTCG		TCGAAGCGTACCAACCAGTC
*PSY*	CCACGCCGAACATGCTTTAG	*ZEP2*	ATACACCGTCTTTGCGGGAG
	GACTTCTTGCACTTGTGCCG		CCATCACCGACATCACTCGT
*PDS1*	TTCTCCACGACACTCAAGGC	*ZEP3*	CGGTTTTTCTGTGCTGGGTG
	CCGGTTTCGATCCAGTCTCC		AGTCTTGAATGGCGGCAGAA
*PDS2*	GTGTTCTCGGTGGCAGTCTT	*VDE*	TTCCATCAAGGCGCAAAAGC
	GAGCCGACGCTAGAGAAGTC		GCTGGGAGGTTTCTCGTTCA
*ZDS*	TTGGACTCGATGGAAGGTGC	*FCPb*	AGCACCGCTTGGATTCTACG
	CCGCTTTCCTCTTTCGCTTG		TGCCAAGTATCCAGCAACGG

### Statistical Analysis

All data were performed in biological triplicates and presented as mean ± standard deviation (SD). Origin V9.0 software was used to plot figures. The statistical analysis was performed by one-way analysis of variance (ANOVA) and LDS *t*-test with SPSS V22.0 software. Significant differentiation level were set at ^∗^*p* < 0.05 and ^∗∗^*p* < 0.01 by compared with control group.

## Results and Discussion

### Effects of Initial Cell Density and Light Intensity

**FIGURE 1:**
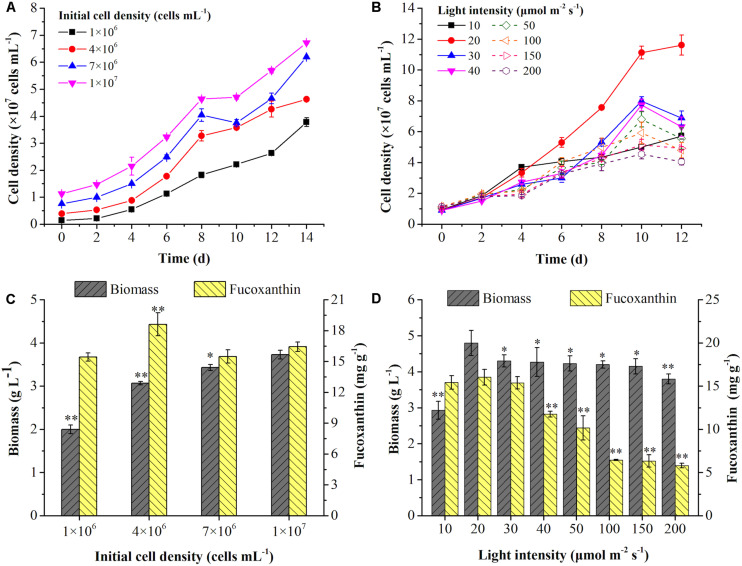
Effects of initial cell density and light intensity on cell growth **(A,B)**, biomass concentration and fucoxanthin content **(C,D)** in the mixotrophic growth of *P. tricornutum*. Significant differentiation level was set at * *p* < 0.05 and ** *p* < 0.01 compared with initial cell density of 1 × 10^7^ cells mL^– 1^ and light intensity of 20 μmol m^– 2^ s^– 1^, respectively.

The effects of initial cell density was shown in Figure 1A. The rapid increase of cell growth were observed in the first 8 days but slowed down in the next 6 days. With the increase of initial cell density, the average specific growth rate of cells was significantly decreased (*p* < 0.01) (Table 2). The highest final cell density (6.72 × 10^7^ cells mL^–1^) and the maximal biomass concentration (3.73 g L^–1^) were achieved at the highest initial cell density (1 × 10^7^ cells mL^–1^) (Figures 1A,C). Even though the highest content (18.61 mg g^–1^) and intercellular amount of Fx (1.23 pg cell^–1^) were attained at 4 × 10^6^ cells mL^–1^ (Figure 1C and Table 2), the highest volumetric concentration of Fx reached to 59.66 mg L^–1^ at the highest initial cell density (1 × 10^7^ cells mL^–1^) (Table 2). Therefore, for improving biomass and Fx production in a shorter time, the highest initial cell density was the option in the following experiments.

**TABLE 2:** Effects of initial cell density and light intensity on cell growth, fucoxanthin production and nutrient consumption in the mixotrophic growth of *P. tricornutum*.

	**μ**(d^–^**^1^)**	**Volumetric Fx**	**Consumption rate (mg L**^–^**^1^d**^–^**^1^)**		**Intercellular Fx**	**Chl *a*(mg g**^–^**^1^)**	**Fx/Chl *a***
						
		**concentration (mg L**^–^**^1^)**			**amount (pg cell**^–^**^1^)**		
			**Glycerol**	**TN**			
**Initial cell density (cells mL**^–^**^1^)**							
1 × 10^6^	0.240.00**	30.840.98**	164.985.42**	4.500.33	0.810.01**	NA	NA
4 × 10^6^	0.180.01**	57.071.66	188.876.35**	7.330.33**	1.230.04**	NA	NA
7 × 10^6^	0.150.00**	53.061.46*	199.873.18*	7.000.17**	0.860.02	NA	NA
1 × 10^7^	0.130.00	59.662.40	220.049.53	9.500.17	0.890.04	NA	NA
**Light intensity (μmol m**^–^**^2^ s**^–^**^1^)**							
10	0.150.00**	45.112.23**	136.114.81**	8.710.38**	0.790.02**	24.630.67*	0.630.04**
20	0.210.01	76.784.26	227.789.62	15.880.13	0.660.01	28.631.55	0.560.01
30	0.170.00*	66.091.97**	166.678.33**	13.001.00**	0.960.04**	25.500.66*	0.600.02*
40	0.160.01**	50.134.35**	155.564.81**	9.320.57**	0.790.03**	16.490.40**	0.710.00**
50	0.140.01**	42.814.62**	119.449.62**	8.170.17**	0.770.02**	14.941.33**	0.680.04**
100	0.120.01**	27.040.89**	141.678.33**	8.500.50**	0.570.04*	8.990.16**	0.720.02**
150	0.130.01**	26.202.13*	122.224.81**	7.830.50**	0.530.03**	7.840.14**	0.810.08**
200	0.110.00**	22.010.46**	144.444.81**	7.590.25**	0.550.03**	7.530.42**	0.770.01**

Data are expressed as mean ± SD of three replicates. NA-No detected. Significant differentiation level with * *p* < 0.05 and ** *p* < 0.01 compared with initial cell density 1 × 10^7^ cells mL^–1^ and light intensity 20 μmol m^–2^ s^–1^, respectively.

To further promote the cell growth under high cell density, the effects of light intensity were evaluated. Interestingly, the increase of light intensity had no obviously negative effect on biomass production from 30 to 200 μmol m^–2^ s^–1^ (Figure 1D), but an inhibiting effect on cell density was observed (Figure 1B). The highest biomass concentration (4.80 g L^–1^) and Fx content (16.03 mg g^–1^) could be reached at light intensity of 20 μmol m^–2^ s^–1^ (Figure 1D). The content and intercellular amount of Fx significantly decreased when the light intensity exceeded 30 μmol m^–2^ s^–1^, which was consist with the previous studies. For instance, the Fx content reached the maximal value at 13.5 μmol m^–2^ s^–1^ (Gomez-Loredo et al., 2016) and dropped from 7.50 mg g^–1^ to 1.10 mg g^–1^ when the light intensity increased from 30 to 180 μmol m^–2^ s^–1^ in *P. tricornutum* (Wang H. et al., 2018). However, the cell growth and biomass production were both decreased with the increase of light intensity under autotrophic condition, which may be explained by the photo-inhibition (Wang H. et al., 2018). Besides, Fx and Chl *a* contents were significantly reduced but fucoxanthin-to-chlorophyll *a* ratio (Fx/Chl *a*) was significantly increased from 20 μmol m^–2^ s^–1^ in this study (*p* < 0.05) (Table 2). Since Chl *a* exists not only in FCP, but also in free Chl *a* (Nymark et al., 2009), the increase of Fx/Chl *a* ratio indicated that free Chl *a* degraded more than that in FCP, resulting in higher degradation of Chl *a* in total Chl *a* than Fx when cells exposed to high light. Similarly, both of Fx and Chl *a* contents decreased sharply with the increase of light intensity in autotrophic *P. tricornutum* (Nur et al., 2019; Conceicao et al., 2020). The transcriptome analysis indicated that the genes encoding enzymes in biosynthesis of GGPP, which is the precursor of both Chl *a* and Fx, were downregulated upon high light intensity in *P. tricornutum*, leading to the reduction of Chl *a* and Fx (Nymark et al., 2009). When cells were exposed to high light, NPQ was activated to convert excess light energy into heat energy (Nymark et al., 2009). In diatoms, Ddx cycle plays a critical role in NPQ, which protects cell from high-light damage (Hao et al., 2018). Under high light, the contents of Ddx and Fx were lower than low light (Conceicao et al., 2020), which due to the upregulation of VDE promoted the conversion of Ddx to Dtx and violaxanthin to zeaxanthin, leading to the decline of Fx (Nymark et al., 2009). Therefore, the dim light at 20 μmol m^–2^ s^–1^ is favorable for cell growth and Fx accumulation under mixotrophic condition without photo inhibition.

### Effects of Light Quality

**FIGURE 2:**
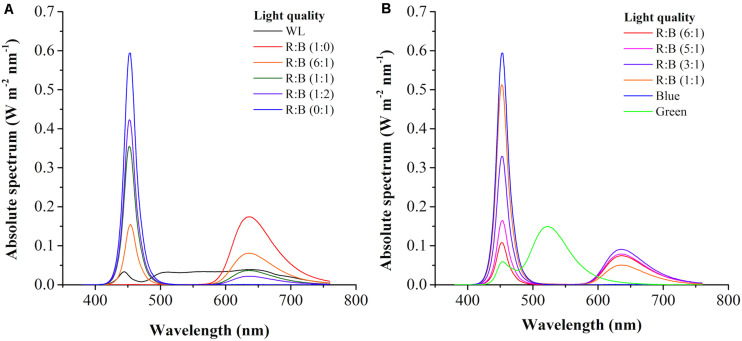
The light spectrum of white light (WL) and various red: blue lights (1:0, 6:1, 1:1, 1:2, 0:1) **(A)** and light regimes in the two-phase culture **(B)**.

**FIGURE 3:**
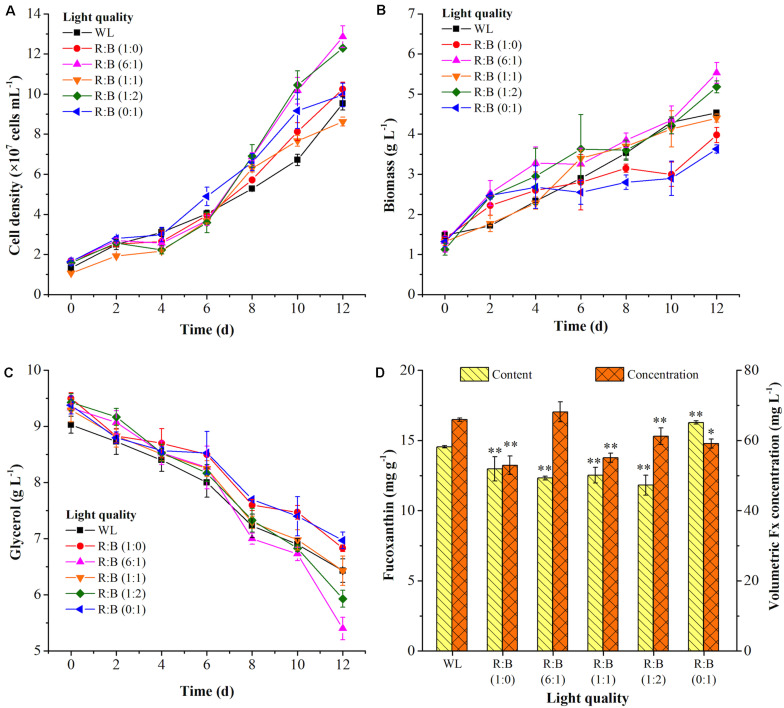
The cell growth **(A)**, biomass **(B)** and glycerol concentration **(C)**, fucoxanthin production **(D)** at white light (WL) and various red: blue lights (1:0, 6:1, 1:1, 1:2, 0:1). Significant differentiation level was set at * *p* < 0.05 and ** *p* < 0.01 compared with white light (WL).

The light spectrums of WL and various red: blue lights (R: B, 0:1, 6:1, 1:1, 1:2, 1:0) were shown in Figure 2A. The wavelength of WL was ranged from 402 nm to 760 nm, and the peak wavelength of blue and red light was 452 nm and 636 nm, respectively. As shown in Figure 3A, the cells grew rapidly under red: blue lights and the highest biomass concentration (5.53 g L^–1^) with productivity (351.39 mg L^–1^ d^–1^) could be achieved at R: B light (6:1), which was 1.22- and 1.38-fold higher than WL (*p* < 0.01) (Figure 3B and Table 3). Even though the biomass productivity in R: B light (6:1) and R: B light (1:2) were similar (Table 3), which might due to the nutritional limitation in the late phase of culture, the former in biomass productivity was 4.12% higher than the later. Similarly, the previous study indicated that the biomass was significantly higher at the mix of red and blue light than the fluorescent WL in autotrophic culture of *P. tricornutum*, but it was similar in the group between R:B (70:30) and R:B (30:70), which was consist with this study (Sirisuk et al., 2018). The increase of biomass in the first 6 days (Figure 3B) was due to the prior uptake of tryptone and urea as carbon and nitrogen source, which explained the slow consumption of glycerol in this period (Figure 3C). The maximum average glycerol consumption rate during 12-days cultivation reached to 327.78 mg L^–1^ d^–1^, while the highest biomass yield per TN consumed (Y_X/TN_) reached to 28.49 mg mg^–1^ at R: B light (6:1) (Table 3). Moreover, the highest Fx content (16.28 mg g^–1^) and Chl *a* content (32.68 mg g^–1^) were attained under BL (Figure 3D and Table 3), which were 12% and 36% higher than WL (*p* < 0.01), respectively. These results were similar with that the Fx and Chl *a* contents were significantly higher under BL than WL in autotrophic *P. tricornutum* and *Coscinodiscus granii* (Sirisuk et al., 2018; Su, 2019). However, the biomass concentration (3.63 g L^–1^) under BL was the lowest one (Figure 3B) in this study. Compared to WL, the results indicated that R: B light (6:1) could promote the cell growth and biomass production, but BL has positive effect on Fx accumulation.

**TABLE 3:** Nutrients consumption, biomass and Fx production at white light and various R: B lights under the mixotrophic mode.

**Light quality**	**μ (d**^–^**^1^)**	**Biomass productivity (mg L**^–^**^1^ d**^–^**^1^)**	**Consumption rate (mg L**^–^**^1^ d**^–^**^1^)**		**Y_X/TN_ (mg mg**^–^**^1^)**	**Chl *a* (mg g**^–^**^1^)**
			**Glycerol**	**TN**		
White light (WL)	0.160.00	254.177.22	216.678.33	11.180.16	22.730.82	23.971.85
R:B (1:0)	0.150.00*	223.612.41**	222.229.62	11.830.17**	18.900.14**	29.280.87*
R:B (6:1)	0.170.00*	351.399.62**	327.784.81**	12.330.17**	28.490.48**	20.530.95*
R:B (1:1)	0.170.00*	256.942.41	238.006.77*	13.750.25**	18.690.50**	22.261.39
R:B (1:2)	0.170.00*	337.508.33**	291.678.33**	12.500.17**	26.450.86**	23.760.46
R:B (0:1)	0.150.01*	193.062.41**	201.396.36	13.170.50**	14.680.58**	32.680.95**

TN, total nitrogen; Y_X/TN_, biomass yield per total nitrogen consumed. Data are expressed as mean ± SD with three replicates. Significant differentiation level with *p < 0.05 and **p < 0.01 compared with white light (WL).

The similar reports could be found that *P. tricornutum* produced more biomass under higher proportion of red light in mixture light under autotrophic condition (Sirisuk et al., 2018), but higher contents of xanthophyll cycle pigments (including Fx) were obtained under BL (Jungandreas et al., 2014). The light qualities, red, green, and blue light, played a vital role in regulation of carbon flow distribution. For example, the shift from BL to red light increased the intermediates of glycolysis and promoted the accumulation of carbohydrates. On the contrary, the shift of red light to BL led to the accumulation of amino acids and tricarboxylic acid (TCA) cycle intermediates, as well as biosynthesis of proteins (Jungandreas et al., 2014). The previous study indicated that the expression of photosynthesis-related nuclear genes were light quality-independent, while the energy transfer efficiency, photo protection and PSII repair related genes were highly dependent on light quality, especially BL (Valle et al., 2014). Similar results were observed in another research, in which compared to red light, BL performed larger pool size of xanthophyll cycle pigments and higher value of NPQ and de-epoxidation state [DES = Ddt/(Ddx + Ddt)], which meant BL showed more capacity of photo protection (Costa et al., 2013). BL not only influenced the genes expression to regulate metabolism, but also directly regulate the activities of specific enzymes, like nitrate reductase (Azuara and Aparicio, 1983). The significant reduction of C/N ratio in cells under BL indicated that it performed higher nitrogen assimilation in cells compared to red light (Jungandreas et al., 2014). This phenomenon was similar in our study, the nitrogen consumption rate was increased with higher proportion of BL in the mixed light except R:B (1:1) (Table 3). For the reason why TN consumption rate at R:B (1:1) was higher than at (1:2), it might be due to that the nitrogen source used in this study was the mixture of tryptone and urea. Perhaps more TN was used as carbon source at R: B (1:1), resulting in the higher TN consumption rate with the lower consumption rate of glycerol in this group. Interestingly, when cells were exposed to WL, BL, and red light, respectively, after dark treatment, the transcript levels of *PSY*, *PDS*, *ZEP*, and *FCPb* increased immediately upon blue and WL, while the expression levels were much lower in response to red light (Coesel et al., 2008). Additionally, transcriptome analysis indicated that the transcripts under BL were enriched in Fx-related expressed sequence tag (EST) database (Coesel et al., 2008), and the expression of *PSY*, *PDS*, *ZDS*, and *ZEP3* were enhanced, resulting in the promotion of Fx production (Costa et al., 2013; Valle et al., 2014). Therefore, choosing R: B light (6:1) as the optimal light in phase 1 and BL as inducing light in phase 2 were carried out in further experiments.

### Effects of Light Shift and Nitrogen Supplementation in Two-Phase Culture

Feeding substrates in batch culture is a common approach in microalgal cultivation which benefits biomass and bioactive compounds production (Ceron-Garcia et al., 2013; Lu et al., 2018). Fx exists in FCP, and sufficient nitrogen is essential not only for biomass production, but also for Fx accumulation (McClure et al., 2018; Wang H. et al., 2018; Nur et al., 2019). Thus, the effects of light shift and nitrogen supplementation in phase 2 were evaluated in two batch cultures.

**FIGURE 4:**
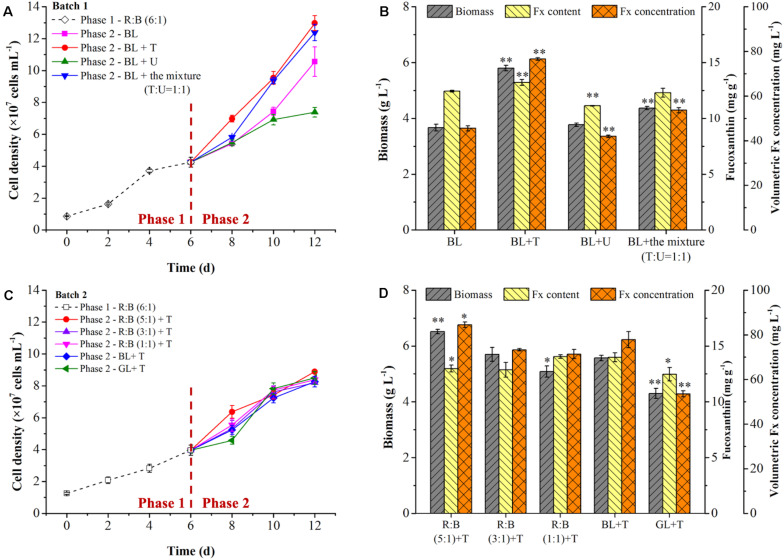
Effects of light shift and nitrogen supplementation on cell growth **(A,C)** and fucoxanthin production **(B,D)** in two phase of two batch cultivation. In batch 1 **(A,B)**, the mixotrophic cells grew at R: B light (6:1) for 6 days in phase 1, then light shifted to pure blue light (BL) with (+) nitrogen supplementation in phase 2. Nitrogen source was presented as urea (U), tryptone (T) and their mixture at a ratio (T:U = 1:1, N mol). Significant differentiation level was set at ** *p* < 0.01 compared with blue light (BL) shift. In batch 2 **(C,D)**, blue light was strengthened solely to form R: B lights (5:1, 3:1, 1:1), or shifted to pure blue light (BL) or pure green light (GL), respectively, with (+) tryptone (T) addition in phase 2. Significant differentiation level was set at * *p* < 0.05 and ** *p* < 0.01 compared with blue light shift with tryptone addition (BL + T) in batch 2.

In the batch 1, the mixotrophic cells were cultivated at R:B light (6:1) for 6 days in phase 1, then light shifted to pure BL for induction with or without supplementation of nitrogen [tryptone, T; urea, U; or the mixture, T:U = 1:1 (N mol)] in different groups in phase 2. As shown in Figure 4A, the culture groups of BL + T or BL+ the mixture in phase 2 could observe the promotion of cell growth compared to BL group, but BL + U inhibited the cell growth as well as Fx production (Figure 4B). The final cell density and biomass concentration reached to the highest value of 1.30 × 10^8^ cells mL^–1^ and 5.80 g L^–1^ by tryptone addition (Figures 4A,B), which were 23% and 58% higher than the control group (BL) (*p* < 0.05), respectively. The highest biomass productivity achieved 373.61 mg L^–1^ d^–1^ under tryptone addition, which was significant higher than BL + U and the control group (Table 4). In contrast, the previous study showed that successive supplementation of 0.01 mol L^–1^ urea in mixotrophic growth of *P. tricornutum* led to 5.37-fold higher biomass, while the biomass was reduced when urea concentration exceeded 0.01 mol L^–1^ with 0.10 mol L^–1^ glycerol (Garcia et al., 2005). This phenomenon was caused by that the cells prioritized to utilize amino acids in tryptone as carbon and nitrogen source in phase 1, while the higher urea residual after urea addition in phase 2 exceed the optimum concentration, leading to the negative impact on cell division and Fx production in this study. As shown in Figure 4B and Table 4, the highest content (13.21 mg g^–1^) and volumetric concentration (76.58 mg L^–1^) of Fx were attained by tryptone addition in phase 2, with the consumption rate of glycerol (233.33 mg L^–1^ d^–1^) and TN (13.69 mg L^–1^ d^–1^). Besides, the intercellular amount of Fx was significantly increased up to 0.61 pg cell^–1^ in BL + T group in phase 2, which was 1.42-fold higher than the control group (BL) (Table 4). Compared to the group of 12-days cultivation under R: B light (6:1) (Figures 3B,D), the biomass, Fx content and volumetric concentration were increased by 5%, 7%, and 12% under the two-phase culture in batch 1 (Figure 4B), respectively.

**TABLE 4:** Effects of LED light shift and nitrogen supplementation in two-phase culture strategy.

**Strategy**	**μ (d**^–^**^1^)**	**Biomass productivity**	**Consumption rate (mg L**^–^**^1^ d**^–^**^1^)**		**Y_X/TN_**	**Intercellular Fx**	**Fx/Chl *a***
						
		**(mg L**^–^**^1^ d**^–^**^1^)**			**(mg mg**^–^**^1^)**	**amount (pg cell**^–^**^1^)**	
			**Glycerol**	**TN**			
**Batch 1: blue light shift with nitrogen supplementation in phase 2**							
BL	0.210.00	208.334.17	144.444.81	11.750.08	17.730.23	0.430.02	0.650.03
BL + T	0.220.00**	373.616.36**	233.3314.43**	13.691.21*	27.412.12**	0.610.03**	0.630.02
BL + U	0.180.00**	201.398.67	180.564.81**	10.810.41*	18.640.14**	0.570.02**	0.670.02
BL + the mixture (T:U = 1:1)	0.220.00**	240.284.81**	238 ± 17.35**	12.220.77	19.701.10*	0.430.01	0.680.01
**Batch 2: light shift with tryptone addition in phase 2**							
R:B (5:1) + T	0.160.00	402.786.36**	386.119.62**	9.330.67	43.272.42**	0.950.02	0.450.05
R:B (3:1) + T	0.160.00	338.8913.39	366.6714.43**	9.670.67	35.111.27	0.890.00*	0.450.01
R:B (1:1) + T	0.160.00	286.116.36**	297.224.81*	9.330.67	30.731.52	0.850.01**	0.430.01
BL + T	0.160.00	338.896.36	275.008.33	10.110.51	33.551.04	0.940.02	0.450.04
GL + T	0.160.00	273.619.62**	247.224.81**	8.780.35*	31.180.61*	0.630.02**	0.620.05*

N, nitrogen; TN, total nitrogen; Y_X/TN_, biomass yield per total nitrogen consumed; Fx, fucoxanthin. U, urea; T, tryptone; T:U = 1:1, the mixture of tryptone and urea; BL, pure blue light; GL, pure green light. Data are expressed as mean ± SD in three replicates. Significant differentiation level with *p < 0.05 and **p < 0.01 compared with the control groups [blue light shift (BL) in batch 1, blue light shift with tryptone addition (BL + T) in batch 2], respectively.

In the batch 2, to verify the inducing effect on Fx production, after cultivated at R: B light (6:1) for 6 days in phase 1, various light shifts were evaluated in phase 2 compared with the control group (BL + T). Among them, BL was enhanced solely to form R: B lights (5:1, 3:1, 1:1), or light shifted to pure green light (GL). All of the groups were combined with tryptone addition in phase 2. The light spectrums were shown in Figure 2B. Interestingly, the cell growth showed obvious difference from the 6th day in phase 2 but reached to a similar level of final cell density (Figure 4C), leading to a same specific growth rate in all groups (Table 4). However, the nutrition consumption rates were different (Table 4), which affected the metabolic flux of nutrients converted to intercellular components (carbohydrates, proteins, lipids etc.) in different groups, resulting in the difference of biomass concentrations. The highest biomass concentration (6.52 g L^–1^), which was 17% higher than the control group (BL + T) (*p* < 0.01), was achieved with the maximum productivity (402.78 mg g^–1^ L^–1^) in R: B (5:1) + T group in phase 2 (Figure 4D and Table 4). Glycerol consumption rate and Y_X/TN_ were consistently increased with the increasing biomass, and reached to the highest value in R: B (5:1) + T group in phase 2 (Table 4), respectively. Compared to the control group (BL + T) in phase 2, even though the Fx content was lower in R:B (5:1) + T group (*p* < 0.05), the intercellular amount of Fx was similar in the two groups (Table 4) but the volumetric concentration of Fx (84.48 mg L^–1^) achieved the highest level (*p* < 0.05) (Figure 4D). Therefore, the shift to R: B (5:1) with tryptone addition in phase 2 was the best option to encourage more biomass production and accumulation of intercellular Fx, leading to an increasing volumetric concentration of Fx.

It is noteworthy that the cell density was increased rapidly in GL + T in phase 2 (Figure 4C), but the lowest biomass concentration was observed (Figure 4D). Similarly in the previous report, the biomass entered stable phase and the lipid content increased by 53% and 29% when light shifted from R:B (50:50) (Sirisuk et al., 2018) and BL (Jung et al., 2019) to green light, respectively. Additionally, the content and intercellular amount of Fx were the lowest in GL + T compared to other groups, resulting in the lowest volumetric concentration of Fx (Figure 4D and Table 4). It was reported that carotenoids content was reduced after 3-days green light exposure (Jung et al., 2019), and the genes encoding LCYB, ZEP1, and ZEP 2 performed high initial transcription levels, then balanced out the difference after 24 h exposure to green light (Valle et al., 2014), which explained the reduction of Fx under green light. Additionally, the previous study indicated that Chl *a* was more inhibited compared to carotenoids under green light induction (Jung et al., 2019). These results explained the increase of Fx/Chl *a* ratio under green light in Table 4. Therefore, shifting to green light was neither the option for biomass nor for Fx production.

### Bioprocess Analysis of R: B (5:1) + T Group in the Batch 2

To further understand the physiological response and regulation mechanism of cells in R: B (5:1) + T group in the batch 2, the biomass and Fx production, nutrient consumption were analyzed in the two-phase culture. The cell samples were taken from the culture in phase 2 at four time points (6th, 8th, 10th, and 12th days) to evaluate the Fx content and key genes expression in Fx biosynthesis pathway.

**FIGURE 5:**
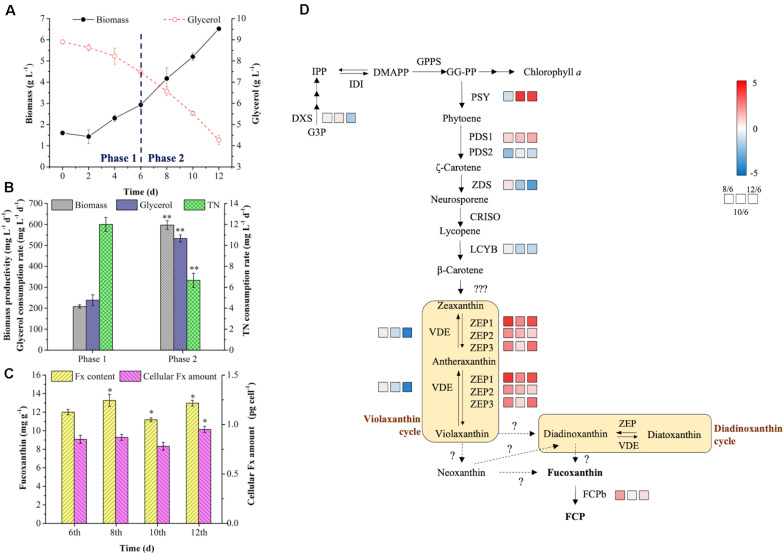
Bioprocess analysis of R: B (5:1) + T group in the batch 2. Production and nutrient consumption **(A,B)** in two-phase culture, fucoxanthin content and intercellular amount **(C)**, genes expression levels in fucoxanthin biosynthesis pathway on the 6th, 8th, 10th, 12th days in phase 2 **(D)**. Significant differentiation level was set with * *p* < 0.05 and ** *p* < 0.01 compared with that at 6th day in phase 1, respectively. The color boxes indicate the expression values of log_2_FC (8th/6th), log_2_FC (10th/6th), and log_2_FC (12th/6th).

As shown in Figure 5A, the biomass increased slowly with low biomass productivity (208.33 mg L^–1^ d^–1^) in phase 1, and then the cells utilized glycerol more quickly for rapid growth from 4th day, which might be due to the exhaustion of available amino acids in tryptone as carbon source of prior utilization. With the rapid consumption of glycerol in phase 2, the biomass productivity reached to 597.22 mg L^–1^ d^–1^, which was 2.87-fold higher than phase 1 (*p* < 0.01) (Figures 5A,B). The glycerol consumption rate achieved 533.33 mg L^–1^ d^–1^ in phase 2, resulting the 223% increase in the final biomass concentration (Figures 5A,B). However, the consumption rate of TN was lower in phase 2 (Figure 5B), which might be caused by a slow urea consumption in the medium since 6th day. The addition of tryptone provided sufficient organic carbon and nitrogen for biomass production in phase 2, while the Fx content and intercellular amount reached to the highest level (13.26 mg g^–1^ and 0.95 pg cell^–1^) on the 8th and 12th day, respectively (Figure 5C). At the end of cultivation, the content and intercellular amount of Fx increased by 8% and 12% compared to day 6 (*p* < 0.05) (Figure 5C). Through enhancing biomass concentration and Fx content in phase 2, the Fx productivity increased to 8.22 mg L^–1^ d^–1^, which was the highest level ever reported so far (Table 5).

**TABLE 5:** Biomass, fucoxanthin content and productivity from *P. tricornutum* in this study compared to the previous literatures.

**Strain**	**Trophicmode**	**Carbon source**	**Nitrogen source**	**Light strategy**	**Biomass(g L**^–^**^1^)**	**Biomass productivity(mg L**^–^**^1^ d**^–^**^1^)**	**Fucoxanthin**		**References**
							**Productivity(mg L**^–^**^1^ d**^–^**^1^)**	**Content(% DW)**	
*P. tricornutum* CCMP 1327	M	0.1 mol L^–1^ glycerol	0.02 mol N L^–1^, mixture (T:U = 1:1), with T addition	Batch 2^∗^	6.52	597.22 (Phase 2)	8.22 (Phase 2)	1.30	This study
*P. tricornutum* SAG 1090-6	M	Spruce hydrolysates (contain 2 g L^–1^ glucose)	Yeast extract (C/N,60)	100 μmol m^–2^ s^–1^, L:D, 14:10 h	3.31	254^#^	–	0.51 (carotenoids)	Patel et al., 2019
*P. tricornutum* UTEX 640	M	0.1 mol L^–1^ glycerol, Semi-continuous culture	0.85 g L^–1^ NaNO_3_	465 μmol m^–2^ s^–1^	12.08	1008	–	0.70 (carotenoids)	Ceron-Garcia et al., 2013
*P. tricornutum* UTEX 640	M	0.1 mol L^–1^ glycerol	0.01 mol L^–1^ urea	165 μmol m^–2^ s^–1^	2.87	396.24^#^	–	0.49 (carotenoids)	Garcia et al., 2005
		0.1 mol L^–1^ glycerol	0.01 mol L^–1^ urea successive implementation	165 μmol m^–2^ s^–1^	15.40	1524^#^	–	–	
*P. tricornutum* CCAP1055/1	A	0.5% CO_2_	0.075 g L^–1^ NaNO_3_	150 μmol m^–2^ s^–1^	<0.2	–	–	2.68	Conceicao et al., 2020
*P. tricornutum* CS-29	A	1% CO_2_	0.75 g L^–1^ NaNO_3_	150 μmol m^–2^ s^–1^	0.37	–	2.16	5.92 ± 2.28	McClure et al., 2018
*P. tricornutum*	A	1% CO_2_	1.45 g L^–1^ KNO_3_	300 μmol m^–2^ s^–1^	4.05 (Day 9)	–	4.73^#^ (Day 6)	1.03 (Day 3) 0.66 (Day 12)	Gao et al., 2017

*M-Mixotrophy; A-autotrophy; T-tryptone. Batch 2^∗^- Phase 1: R:B light (6:1) at 20 μmol m^–2^ s^–1^ for 6 days; Phase 2: R:B light (5:1) at 25 μmol m^–2^ s^–1^ for 6 days. ^#^-maximum biomass/fucoxanthin productivity during the cultivation.*

It is known that biosynthesis of Fx involved in methylerythritol phosphate (MEP) pathway, IPP pathway and Fx formation (Bertrand, 2010). However, the final steps of Fx formation were not know completely so far (Lohr and Wilhelm, 2001; Dambek et al., 2012; Figure 5D). The DXS and PSY are two key rate-limiting enzymes to control the biosynthesis of Fx. The previous study indicated that the overexpression of *DXS* and *PSY* could raise the content of Fx by 2.40- and 1.80-fold in *P. tricornutum*, respectively (Eilers et al., 2016). Under the mixotrophic condition, glycerol consumption could supply abundant G3P (Villanova et al., 2017), which is the substrate of DXS and carbon skeleton of carotenoids biosynthesis. In this study, the expression of *DXS* was almost stable from 6th to 10th day, but decreased 1.46-fold on the 12th day compared to the 6th day (Figure 5D), suggesting that the BL strengthening with tryptone addition induced more carbon flux to TCA cycle and protein biosynthesis rather than pigments formation (Jungandreas et al., 2014). In contrast, the expression of *PSY* was 0.70-fold downregulated on the 8th day and significantly upregulated (|log_2_fold change| > 2, *p* < 0.01) from the 10th day. Similarly, the previous report indicated that BL induction was proved to upregulate the expression of *PSY* under autotrophic condition in *P. tricornutum* (Coesel et al., 2008). One possible reason for the delay of *PSY* response was that the cells need time to adapt to light shift (Jungandreas et al., 2014), the other possibly reason relate to the expression of genes encoding ZEP. There were three types of *ZEP* identified in *P. tricornutum* (Bowler et al., 2008), and the expression levels of *ZEP1*, *ZEP2*, and *ZEP3* were 4.87-, 2.06-, and 2.28-fold upregulated on the 8th day compared to 6th day, which not only contributed to the improvement of Fx content on the 8th day, but also accelerated the conversion from zeaxanthin to violaxanthin, leading to the promotion of *PSY* expression level from 10th day. Additionally, the expression level of *FCPb* was 1.70-fold higher on the 8th day than the 6th day but returned to the initial level from 10th day. However, the expression of *VDE* performed an opposite pattern, in which *VDE* transcript level changed slightly from 6th to 10th day and was 4.29-fold decreased on the 12th day (*p* < 0.01). In the previous report, the accumulation of Fx was not synchronized with the abundance of *PSY* transcripts, while the Fx content at the stationary phase was correlated with the amounts of *PSY* transcripts at the exponential phase (Kadono et al., 2015). Therefore, even though the intercellular amount of Fx increased slightly on the 8th day, the Fx content on the 8th day in phase 2 was significantly improved which depended on the upregulation of *ZEP*s and *FCPb*. And the reduction of intercellular amount and content of Fx on the 10th day might be due to the downregulation of *PSY* on the 8th day and to initial transcript level of *FCPb* on the 10th day. More importantly, the continued upregulation of *PSY* and *ZEP*s with downregulation of *VDE* contributed to the final Fx accumulation (both of intercellular amount and content) on the 12th day (Figure 5C), which proved that the option of R: B (5:1) + T in phase 2 was beneficial for enhancing Fx production in two-phase culture in the batch 2.

It was noteworthy that the expression of *ZEP*s were significantly upregulated (| log_2_fold change| > 2, *p* < 0.01) when the culture shifted to phase 2. Among them, *ZEP1* was the most sensitive gene in response to the BL induction (Figure 5D). A similar phenomenon was observed in the previous study, in which the increase of *ZEP1* transcript level was over 50-fold higher than *ZEP2* and *ZEP3* under BL induction after dark treatment (Coesel et al., 2008). Since violaxanthin cycle and Ddx cycle were two xanthophyll cycles in diatoms participated in NPQ (Lavaud et al., 2003), BL and light intensity play vital roles in NPQ (Bertrand, 2010; Costa et al., 2013). One possible explanation was that *P. tricornutum* did not have the specific enzymes of Dtx epoxidase/diadinoxanthin de-epoxidase (DEP/DDE) in Ddx cycle, and the enzymes of ZEP/VDE in violaxanthin cycle played the same role instead (Bowler et al., 2008). So the *ZEP1* regulated the transformation from zeaxanthin to violaxanthin and *ZEP3* regulated the conversion from Dtx to Ddx (Nymark et al., 2009), leading to different response pattern of the *ZEP1* and *ZEP3* under BL. The another possible explanation was that *ZEP1* and *ZEP2* were suggested to be classified into category that contained light-harvesting complex and enzymes for pigments synthesis, while *ZEP3* was classified as enzyme involved in photo-protection (Nymark et al., 2013; Valle et al., 2014). In this study, the high cell density (Figure 4C) resulted in less light exposure to individual cell during the 6th to 12th day, and *ZEP3* might drive the conversion of violaxanthin to zeaxanthin and Ddx to Dtx in low light.

In a word, the expression levels of key genes involving Fx biosynthesis (*PSY*, *ZEP*s, *FCPb*, and *VDE*) were significantly regulated by BL strengthening and tryptone addition, which had positive effects on Fx accumulation, leading to the a great improvement of Fx production.

## Conclusion

In this study, the combination of red: blue light at a favorable ratio in phase 1 and light shift with tryptone addition in phase 2 was employed to significantly improve Fx production by the mixotrophic *P. tricornutum*, which achieved the highest level of ever reported so far. The analysis of gene expression levels involving Fx biosynthesis revealed that *PSY*, *ZEP*s, and *FCPb* were upregulated while *VDE* was downregulated under BL strengthening and tryptone addition, indicating the possible regulatory mechanism on the enhanced Fx production in phase 2. This study developed a novel approach of two-phase culture to produce Fx efficiently by the mixotrophic *P. tricornutum*, which facilitate the scale-up production of Fx by photo fermentation in the future.

## Data Availability Statement

All datasets presented in this study are included in the article.

## Author Contributions

RY and DW conceived and designed the experiments and drafted the manuscript. RY performed the experiments and analyzed the data. DW contributed to the funding. All authors contributed to the article and approved the submitted version.

## Conflict of Interest

The authors declare that the research was conducted in the absence of any commercial or financial relationships that could be construed as a potential conflict of interest.

## Footnotes

1 https://www.kegg.jp/kegg-bin/show_organism

2 https://www.ncbi.nlm.nih.gov/tools/primer-blast/

## Abbreviations

Chl*a*: chlorophyll *a*
CRISO: carotenoid isomerase
Ddx: diadinoxanthin
DMAPP: dimethylallyl diphosphate
Dtx: diatoxanthin
DXS: 1-deoxy-D-xylulose 5-phosphate synthase
FCP: fucoxanthin-chlorophyll-protein
Fx: fucoxanthin
G3P: glyceraldehyde-3-phosphate
GGPP: geranylgeranyl diphosphate
GPPS: geranyl pyrophosphate synthase
IDI: isopentenyl diphosphate: dimethylallyl diphosphate isomerase
IPP: isopentenyl pyrophosphate
LCYB: lycopene β-cyclase
PDS: phytoene desaturase
PSY: phytoene synthase
VDE: violaxanthin de-epoxidase
ZDS: ζ-Carotene desaturase
ZEP: zeaxanthin epoxidase.
